# *Brassica rapa* L. Polysaccharides Alleviate Cyclophosphamide-Induced Intestinal Mucosal Injury in Mice by Modulating Oxidative Stress, Immune Responses, and Gut Microbiota

**DOI:** 10.3390/microorganisms14051146

**Published:** 2026-05-19

**Authors:** Xiaolong Cao, Xiangrui Zhu, Hao Lin, Lingyu Guo, Hua Shui, Zhen Wang, Enci Shen, Zegao Guo, Ruizhe Zhang, Xin Li

**Affiliations:** Xinjiang Production and Construction Corps Key Laboratory of Oasis Town and Mountain-Basin System Ecology, Key Laboratory of Xinjiang Phytomedicine Resource Utilization, Ministry of Education, College of Life Sciences, Shihezi University, Shihezi 832003, China; lpqjzk996di0@163.com (X.C.); zhuxiangrui@xjshzu.com (X.Z.); shayouxiangdizhiya@163.com (H.L.); gjy13199875537@163.com (L.G.); 18993110505@163.com (H.S.); 15225877726@163.com (Z.W.); 15655231690@163.com (E.S.); 18196939356@163.com (Z.G.); 15534529193@163.com (R.Z.)

**Keywords:** *Brassica rapa* L. polysaccharides, cyclophosphamide, gut microbiota, immune regulation, intestinal injury, oxidative stress

## Abstract

This study systematically evaluated the modulatory effects of *Brassica rapa* L. polysaccharides (BRP) on intestinal mucosal injury in a CTX-induced mouse model. The results showed that high-dose BRP (HBRP) significantly alleviated oxidative damage, with CAT activity increased by approximately 2-fold, SOD increased by 1.3-fold, GSH-Px increased by 70%, and MDA levels decreased by 65%. Meanwhile, liver injury was improved, as ALT and AST decreased by 35% and 55%, respectively. CTX markedly suppressed immune function, while BRP intervention significantly restored cytokine levels, with IL-1β increased by up to 2.5-fold, TNF-α by 1.3-fold, and IL-4 by 2.1-fold. In addition, BRP significantly modulated gut microbiota composition. The number of unique ASVs decreased from 316 in the normal control (NC) group to 63 in the model control (MC) group and recovered to 140 in the HBRP group. At the phylum level, Bacillota increased from 55% to 97% (MC) and decreased to 65% after BRP intervention, while Bacteroidota recovered to 25%. At the genus level, *Candidatus_Arthromitus* decreased from 40% to nearly 0%, whereas beneficial bacteria such as *Ligilactobacillus* and norank_f__Muribaculaceae were restored. Overall, BRP effectively alleviates CTX-induced intestinal injury in a dose-dependent manner through antioxidant, immunomodulatory, and gut microbiota–regulating mechanisms.

## 1. Introduction

*Brassica rapa* L., a biennial herbaceous plant belonging to the Brassicaceae family [1], has long been used in traditional Uyghur medicine for the treatment of respiratory ailments such as cough and asthma, reflecting its important ethnomedicinal value and potential pharmacological properties [2]. This plant is rich in bioactive compounds such as glucosinolates, flavonoids, anthocyanins, terpenes, and coumarins, as well as antioxidants including vitamin C, vitamin E, carotenoids, and antioxidant enzymes [3,4,5]. These components contribute to diverse health benefits, including antioxidative, immunomodulatory, and anticancer effects [6,7,8]. Among them, *Brassica rapa* L. polysaccharides (BRP) have been identified as key functional constituents [9,10,11]. However, their protective role against chemotherapy-induced intestinal mucosal injury, particularly through modulation of gut microbiota and mucosal repair mechanisms, remains insufficiently understood.

Cyclophosphamide (CTX), a widely used alkylating chemotherapeutic and immunosuppressive agent, is known for its efficacy in treating various malignancies but is also associated with severe adverse effects [12]. In addition to its cytotoxic action on tumor cells, CTX causes substantial damage to rapidly proliferating intestinal epithelial cells, leading to disruption of villus and crypt architecture, increased intestinal permeability, and excessive oxidative stress and inflammatory responses [13,14]. These changes not only impair intestinal barrier function but also increase susceptibility to infections and systemic complications [15]. Moreover, CTX has been reported to disturb gut microbiota composition, promoting the overgrowth of pathogenic bacteria such as *E. coli* and *Shigella*, thereby exacerbating intestinal dysfunction [16]. Therefore, identifying safe and effective natural agents to mitigate CTX-induced intestinal injury is of significant clinical importance.

The intestine serves not only as a digestive organ but also as a critical immune organ, maintaining host homeostasis through a complex network involving the intestinal epithelium, immune system, and microbiota [17,18,19]. The intestinal barrier is composed of various immune cells, including T cells, B cells, innate lymphoid cells, and macrophages, which collectively regulate immune responses [20]. When the barrier is disrupted, immune signaling pathways such as NF-κB are activated, leading to cytokine release and subsequent immune cell differentiation, which play roles in mucosal repair [21,22]. Additionally, the intestinal microenvironment, including oxygen availability and cellular metabolism, is closely linked to microbial colonization. Factors such as IL-4 and microbial metabolites like butyrate can regulate metabolic pathways and promote a hypoxic environment favorable for beneficial anaerobic bacteria, thereby maintaining microbial balance [23]. Disruption of the gut microbiota–immune interaction can contribute to various diseases, highlighting the importance of maintaining intestinal homeostasis.

In recent years, increasing attention has been paid to the polysaccharides derived from edible and medicinal plants as potential therapeutic agents due to their safety, availability, and diverse biological activities [24]. They modulate immune responses by directly acting on immune cells, improving gut microbiota composition, and promoting the production of short-chain fatty acids (SCFAs) [25,26]. For instance, plant-derived polysaccharides such as *Glycyrrhiza* polysaccharide (GP), *Ephedra sinica* polysaccharide (ESP), and *Lycium barbarum* polysaccharide (LBP) can stimulate cytokine production, increase the abundance of SCFA-producing bacteria, and enhance intestinal mucosal immunity [27,28,29]. However, the regulatory effects of BRP on CTX-induced small intestinal mucosal injury and gut microbial diversity have not been fully elucidated. In this study, we systematically investigated the protective effects of BRP in a CTX-induced mouse model by integrating histological, biochemical, and microbiological analyses, including assessments of oxidative stress, inflammatory responses, and gut microbiota composition. Our findings will provide a reference for developing BRP as a potential adjuvant intervention to alleviate CTX-induced intestinal mucosal injury.

## 2. Materials and Methods

### 2.1. Preparation of Brassica rapa *L.* Polysaccharides (BRP)

*Brassica rapa* L. tubers were cut into uniformly sized pieces, crushed by a grinder (Jiangsu Cayoudi Medical Instrument Co., Ltd., Yixing, China), and then soaked in 85% ethanol overnight. After centrifugation, the precipitate was collected. The precipitate was subjected to ultrasonic disruption, followed by soaking in distilled water overnight and subsequent centrifugation to obtain the supernatant. Absolute ethanol was added to the supernatant to a final concentration of 90% for ethanol precipitation overnight. After centrifugation, the precipitate was collected to obtain crude polysaccharides. The crude polysaccharides were dissolved in a small amount of water to obtain a concentrated solution [30]. The Sevage methodwas then used to remove proteins, resulting in a crude polysaccharide solution. The polysaccharide content was determined by the sulfuric acid-phenol method. The obtained crude polysaccharide solution was purified using macroporous resin. Specifically, D101 macroporous resin was selected and soaked in 5% HCl for 4 h, then washed with distilled water to neutrality. This was followed by soaking in 5% NaOH for 4 h and washing with distilled water to neutrality. Finally, the resin was soaked in absolute ethanol for 24 h and then washed with distilled water until no ethanol odor remained, completing the activation process [31]. A column was wet-packed with 10 mL of the prepared macroporous resin using distilled water (pH = 4). A polysaccharide solution (3 mg/mL) was loaded onto the column at a flow rate of 2.5 BV/h. After the macroporous adsorption resin was fully saturated, the column was rinsed with distilled water. Elution was then performed using 3 bed volumes (BV) of an 80% ethanol solution at a desorption rate of 2 BV/h. The eluate was collected and freeze-dried to obtain the *Brassica rapa* L. polysaccharide (BRP) powder.

### 2.2. Animal Experimental Design

This study was approved by the Ethical Review Approval Letter from the Bioethics Committee of Shihezi University (Ethical Approval No. A2024-142) and was conducted in accordance with the Guide for the Care and Use of Laboratory Animals (8th edition) established by the NIH. Specific pathogen-free (SPF) KM mice were housed in stainless steel cages under standardized environmental conditions, including a controlled ambient temperature of 25 ± 2 °C, relative humidity maintained between 50% and 75%, and a 12 h light/12 h dark photoperiod.

Dose selection rationale: The doses of BRP (80 mg/kg for LBRP, 200 mg/kg for HBRP) were selected based on the following considerations: (1) Preliminary dose-finding experiments in our laboratory indicated that BRP at 80 mg/kg exerted moderate protective effects against CTX-induced immunosuppression, while 200 mg/kg showed more pronounced efficacy without any observable toxicity; (2) The two-dose design (low and high) was intended to evaluate dose-dependent effects of BRP. Animals were provided ad libitum access to a standard laboratory rodent diet and sterile drinking water throughout the study. Following an 8-day acclimatization period to minimize environmental stress and ensure physiological stabilization, a total of 40 mice were randomly allocated into four experimental groups (*n* = 10 per group): the normal control group (NC), the cyclophosphamide-treated model group (MC), the low-dose BRP treatment group (LBRP), and the high-dose BRP treatment group (HBRP). Randomization was performed to reduce selection bias and ensure comparable baseline characteristics among groups. The specific dosing regimen and administration protocol are summarized in Table 1. All treatments were administered via oral gavage once daily for the designated experimental period.

Body weight was measured and recorded at 2-day intervals to monitor general health status and evaluate treatment-related effects. Animals were observed daily for signs of distress, abnormal behavior, or adverse reactions. On day 17 of the experimental protocol, mice were subjected to a 12-h fasting period with free access to water prior to sacrifice to minimize the influence of recent food intake on biochemical and histological parameters. Subsequently, animals were humanely euthanized by cervical dislocation under ethical approval.

Immediately after euthanasia, the abdominal cavity was aseptically opened, and the duodenum was carefully excised. The duodenal tissue was segmented according to downstream analytical requirements, including histopathological examination and biochemical assays. Blood samples were collected to obtain serum, and duodenal luminal contents were simultaneously harvested under sterile conditions. All collected samples were promptly processed or stored at −80 °C until further analysis to preserve biological integrity.

### 2.3. Small Intestine H&E Staining

The small intestine was carefully excised immediately after sacrifice and gently rinsed with cold physiological saline to remove residual luminal contents. The tissue samples were then immersed in freshly prepared 4% (*w*/*v*) paraformaldehyde solution and fixed for a minimum of 24 h at room temperature to ensure adequate preservation of cellular morphology and tissue architecture. Following fixation, the specimens were processed through a graded ethanol dehydration series, sequentially immersed in 80%, 90%, and 95% ethanol solutions to gradually remove water from the tissues. Complete dehydration was achieved with absolute ethanol prior to clearing. The dehydrated samples were subsequently cleared in xylene to replace the ethanol and enhance tissue transparency, facilitating paraffin infiltration [32]. After clearing, the tissues were embedded in molten paraffin wax under controlled conditions to obtain uniform paraffin blocks. Once solidified, the paraffin-embedded samples were sectioned into approximately 4–5 μm thick slices using a rotary microtome (Leica RM2235, Leica Biosystems, Wetzlar, Germany). The sections were mounted onto glass slides and dried to promote adhesion.

For histopathological evaluation, the sections were deparaffinized in xylene, rehydrated through a descending ethanol gradient, and subjected to Hematoxylin and Eosin (H&E) staining [33]. Hematoxylin was used to stain cell nuclei a deep blue-purple color, while eosin counterstained the cytoplasm and extracellular matrix in varying shades of pink, thereby clearly delineating tissue structures. Stained sections were examined and photographed under a light microscope (Leica DMi8, Leica Microsystems, Wetzlar, Germany). Morphological parameters of the intestinal mucosa, including villus height, crypt depth, epithelial integrity, and inflammatory cell infiltration, were evaluated to assess histological alterations among the experimental groups.

### 2.4. Liver Biochemical Indicators

Fresh liver tissues were rapidly excised following euthanasia and immediately rinsed with ice-cold physiological saline to remove residual blood and impurities. The tissues were blotted dry, accurately weighed, and then homogenized in pre-chilled phosphate-buffered saline (PBS) or an appropriate extraction buffer at a specified tissue-to-buffer rati using a glass-Teflon homogenizer (Wheaton, Millville, NJ, USA) under ice-bath conditions to prevent enzymatic degradation and oxidative alterations during processing. The resulting homogenates were centrifuged at 4 °C and 3000 rpm for 10 min to remove cellular debris and insoluble materials. After centrifugation, the supernatants were carefully collected for subsequent biochemical analyses. All procedures were performed at low temperature to preserve enzyme activity and maintain sample stability.

The activities of antioxidant enzymes, including catalase (CAT), superoxide dismutase (SOD), and glutathione peroxidase (GSH-Px), were determined to evaluate hepatic antioxidant defense capacity. The level of malondialdehyde (MDA), an indicator of lipid peroxidation, was measured to assess oxidative damage to cellular membranes. In addition, alanine aminotransferase (ALT) and aspartate aminotransferase (AST) activities were quantified as sensitive biomarkers of hepatocellular injury and liver function status. All biochemical parameters were analyzed using commercially available assay kits in strict accordance with the manufacturer’s instructions. The kits were obtained from Nanjing Jiancheng Bioengineering Institute (Nanjing, China) [34]. Absorbance values were measured using a microplate reader (Thermo Fisher Scientific, Waltham, MA, USA) at the specified wavelengths, and enzyme activities or metabolite concentrations were calculated based on standard curves or formulae provided in the kit protocols. Results were normalized to protein concentration where applicable to ensure comparability among samples.

### 2.5. Serum ELISA Analysis

Whole blood samples were collected from mice under sterile conditions and immediately transferred into 1.5 mL sterile microcentrifuge tubes without anticoagulant. The tubes were maintained in a slightly inclined position at room temperature for approximately 30 min to allow complete coagulation. This step facilitated efficient clot retraction and subsequent serum separation. After clot formation, the samples were centrifuged at 2500 rpm for 10 min (typically at 4 °C to preserve protein stability). Following centrifugation, the clear supernatant (serum) was carefully aspirated to avoid disturbing the blood clot and transferred into new sterile tubes [35]. The collected serum samples were either analyzed immediately or stored at −80 °C for subsequent cytokine quantification.

Serum levels of pro-inflammatory and anti-inflammatory cytokines, including interleukin-1α (IL-1α), interleukin-1β (IL-1β), interleukin-4 (IL-4), interleukin-6 (IL-6), interleukin-10 (IL-10), tumor necrosis factor-α (TNF-α), and interferon-γ (IFN-γ) were determined using commercially available enzyme-linked immunosorbent assay (ELISA) kits. The kits were purchased from Suzhou Grace Biotechnology Co., Ltd. (Suzhou, China) and used strictly according to the manufacturer’s instructions. Briefly, serum samples and serially diluted standards were added to microplate wells pre-coated with specific monoclonal antibodies against the target cytokines. After incubation, unbound substances were removed by washing, and enzyme-conjugated secondary antibodies were added to form antigen-antibody complexes. Subsequently, a chromogenic substrate solution was introduced, and color development was proportional to the cytokine concentration present in the sample [36]. The reaction was terminated using a stop solution, and absorbance was measured at the recommended wavelength (typically 450 nm) using a Multiskan GO manufactured by Thermo Fisher Scientific (Waltham, MA, USA).

Cytokine concentrations were calculated by plotting a standard curve generated from known concentrations of recombinant cytokine standards and applying a four-parameter logistic regression model. All samples were analyzed in duplicate to ensure reliability, and intra-assay variability was controlled according to kit specifications.

### 2.6. 16S rRNA Gene Sequencing Analysis

Primer trimming: Forward and reverse primer sequences were removed from the demultiplexed reads using the cutadapt plugin in QIIME2 prior to denoising, allowing a maximum error of 0 bases. The paired-end raw sequences obtained from sequencing were subjected to quality control and assembly, followed by sample demultiplexing based on barcode and primer sequences. Subsequently, denoising was performed using DADA2 (version 1.0.3) to obtain high-precision amplicon sequence variants (ASVs), from which non-target sequences originating from chloroplasts and mitochondria were removed [37]. Rarefaction depth: To normalize sampling effort, all samples were rarefied to 20,000 sequences per sample, which retained an average Good’s coverage of 99.09%. Taxonomic annotation of ASVs was conducted using the Silva database (version 138), and the functional potential of microbial communities was predicted using PICRUSt2 (version 2.4.1). All the above analytical procedures were performed on the Majorbio Cloud Platform (https://cloud.majorbio.com).

Total genomic DNA was extracted from duodenal contents using a commercial microbial DNA isolation kit with mechanical and chemical lysis. DNA quality was assessed by spectrophotometry and agarose gel electrophoresis. The V3-V4 region of the 16S rRNA gene was amplified by PCR using primers.

338F (5′-ACTCCTACGGGAGGCAGCAG-3′)

806R (5′-GGACTACHVGGGTWTCTAAT-3′)

Purified PCR products were pooled in equimolar concentrations and subjected to paired-end sequencing (2 × 300 bp) on the Illumina MiSeq platform (Illumina, San Diego, CA, USA). The raw sequencing data have been deposited in the NCBI Sequence Read Archive (SRA) under BioProject accession number PRJNA1462805. Raw sequencing data were quality-filtered, merged, and processed to remove chimeric sequences. Amplicon sequence variants (ASVs) were generated depending on the bioinformatic pipeline. Taxonomic classification was performed using a reference database such as SILVA.

### 2.7. Analysis of Differentially Abundant Taxa

Linear discriminant analysis (LDA) effect size (LEfSe) analysis was performed to identify bacterial taxa that differed significantly in relative abundance from the phylum to genus level among groups using the online tool available at (http://huttenhower.sph.harvard.edu/LEfSe (accessed on 16 November 2025)). Taxa with an LDA score (log10) > 2.0 and a *p* < 0.05 (Kruskal-Wallis test followed by Wilcoxon rank-sum test for pairwise comparisons) were considered differentially abundant.

### 2.8. Data Statistics

Experimental data were preliminarily organized using Excel software. One-way analysis of variance (ANOVA) and graph plotting were performed using GraphPad Prism (version 9.0.0). Data are expressed as mean ± standard deviation (mean ± SD). Prior to analysis, the normality of all data distributions was assessed using the Shapiro-Wilk test, and homogeneity of variances was verified using Levene’s test. Data met the assumptions of normality and homoscedasticity. Statistical analyses were performed using GraphPad Prism (version 9.0.0). One-way analysis of variance (One-way ANOVA) was used to compare differences among multiple groups, followed by Duncan’s multiple range test for post-hoc comparisons. Statistical significance was defined as * *p* < 0.05, ** *p* < 0.01, and *** *p* < 0.001, respectively. Different lowercase letters indicate statistically significant differences between groups (*p* < 0.05), while the same letter indicates no statistically significant difference (*p* > 0.05).

## 3. Results

### 3.1. BRP Ameliorated CTX-Induced Intestinal Structural Damage

**Figure 1:**
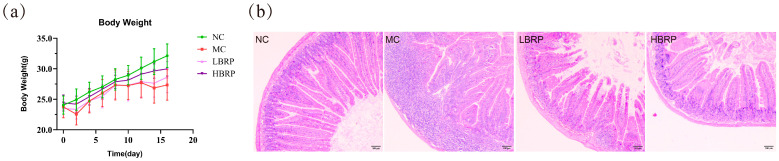
BRP ameliorated CTX-induced intestinal structural damage (**a**) Mouse Body Weight Change Curve. (**b**) Representative image of H&E-stained pathological section of Jejunum tissue (200×). NC (Normal Control), MC (Model Control), LBRP (low-dose BRP group), HBRP (high-dose BRP group).

During the experimental period, the body weight of the mice exhibited fluctuations. The body weight changes of the four groups, NC (normal control group), MC (model control group), LBRP (low-dose BRP group), and HBRP (high-dose BRP group), were monitored over a 17-day period. The HBRP group showed a clear upward trend, with its body weight curve, particularly during and after the polysaccharide administration phase, approaching the level of the NC group (Figure 1a). It is noteworthy that during both the polysaccharide administration phase and the recovery period, the HBRP group exhibited a trend of increased body weight compared to the MC group. Subsequent histological analysis via H&E staining provided further insights (Figure 1b). The intestinal tissue of mice in the model group (MC) exhibited extensive immune cell infiltration and villus structural disorganization, indicating that CTX successfully induced intestinal inflammatory responses and tissue injury. In contrast, no obvious immune cell infiltration was observed in the intestinal tissues of the low- and high-dose BRP intervention groups (LBRP and HBRP), and the villus structure remained relatively intact, demonstrating that *Brassica rapa* L. polysaccharides can effectively alleviate CTX-induced intestinal tissue damage.

### 3.2. Effects of BRP on Liver Physiological and Biochemical Parameters and Serum Immunological Indicators

**Figure 2:**
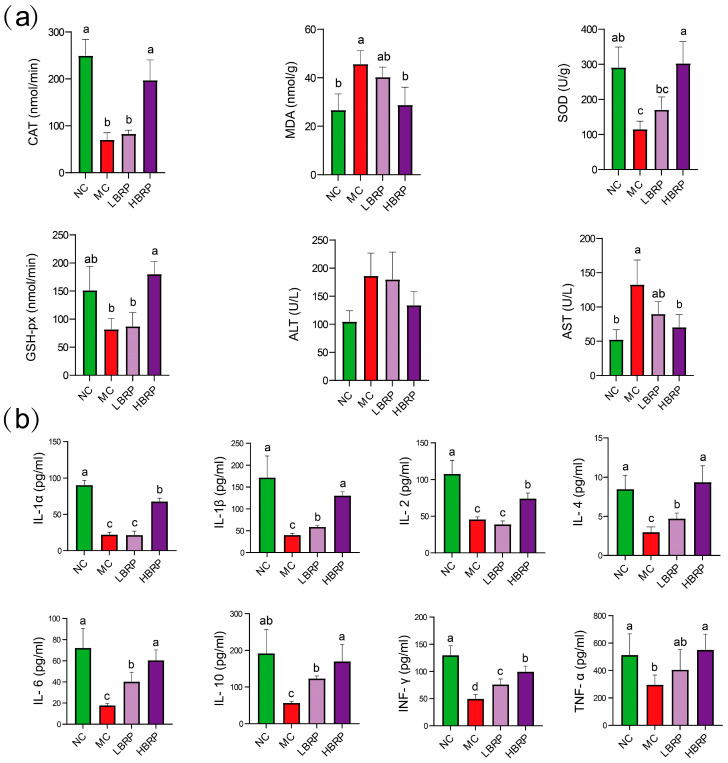
BRP Ameliorates Hepatic Injury in an Experimental Model by Alleviating Oxidative Stress and Modulating Inflammatory Cytokine Levels. (**a**) Comparison of oxidative stress and liver injury-related indicators among experimental groups. CAT: Catalase; MDA: Malondialdehyde; SOD: Superoxide Dismutase; GSH-Px: Glutathione Peroxidase; ALT: Alanine Aminotransferase; AST: Aspartate Aminotransferase. (**b**) Comparison of inflammatory cytokine levels among experimental groups. IL-1α/1β: Interleukin-1alpha/1beta; IL-2: Interleukin-2; IL-4: Interleukin-4; IL-6: Interleukin-6; IL-10: Interleukin-10; IFN-γ: Interferon-gamma; TNF-α: Tumor Necrosis Factor-alpha. Respectively, Different lowercase letters indicate statistically significant differences between groups (*p* < 0.05), while the same letter indicates no statistically significant difference (*p* > 0.05).

CTX administration markedly disrupted the oxidative balance in mice. Compared with the normal control (NC) group, the model control (MC) group exhibited significantly reduced activities of antioxidant enzymes, including catalase (CAT), superoxide dismutase (SOD), and glutathione peroxidase (GSH-Px) (*p* < 0.05). In parallel, malondialdehyde (MDA) levels were significantly elevated in the MC group, indicating enhanced lipid peroxidation and oxidative stress. Supplementation with BRP significantly ameliorated CTX-induced oxidative stress. Both low-dose (LBRP) and high-dose (HBRP) treatments increased CAT, SOD, and GSH-Px activities while reducing MDA levels compared with the MC group. Notably, the HBRP group showed antioxidant enzyme activities comparable to or exceeding those of the NC group, suggesting a dose-dependent protective effect. In addition, CTX treatment resulted in marked liver function impairment, as evidenced by significantly elevated serum alanine aminotransferase (ALT) and aspartate aminotransferase (AST) levels in the MC group relative to the NC group (*p* < 0.05). BRP supplementation effectively reduced ALT and AST levels, with the HBRP group showing a more pronounced improvement (Figure 2a), indicating alleviation of CTX-induced hepatic injury.

CTX administration significantly suppressed serum cytokine production. Levels of multiple immune-related cytokines, including IL-1α, IL-1β, IL-2, IL-4, IL-6, IL-10, IFN-γ, and TNF-α, were markedly decreased in the MC group compared with the NC group (*p* < 0.05), reflecting CTX-induced immune dysfunction. BRP intervention significantly restored cytokine levels in a dose-dependent manner. Both LBRP and HBRP groups exhibited increased concentrations of pro-inflammatory and anti-inflammatory cytokines compared with the MC group. In particular, HBRP treatment resulted in significantly higher levels of IL-1β, IL-2, IL-6, IL-10, IFN-γ, and TNF-α, approaching or reaching levels observed in the NC group (Figure 2b). These results indicate that BRP effectively counteracts CTX-induced immune suppression and promotes recovery of immune function.

### 3.3. Effects of BRP on Sequencing Depth and Gut Microbial Community Structure

**Figure 3:**
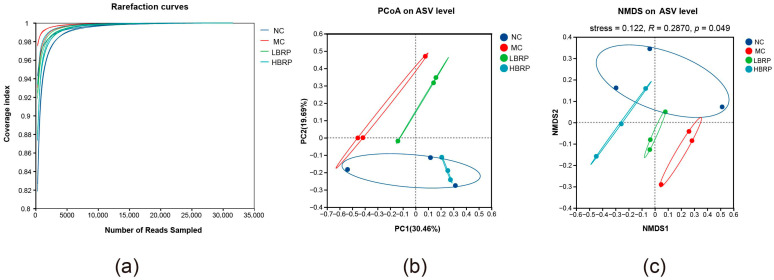
Effects of BRP on sequencing depth and gut microbial community structure at the ASV level. (**a**) Rarefaction curves showing sequencing depth and coverage of gut microbiota samples in different groups. (**b**) Principal coordinates analysis (PCoA) based on ASV-level Bray-Curtis distances. (**c**) Non-metric multidimensional scaling (NMDS) analysis of gut microbial communities among different groups. NC (Normal Control), MC (Model Control), LBRP (low-dose BRP group), HBRP (high-dose BRP group).

To assess the sufficiency of sequencing data and the overall structural differences in intestinal microbiota across different treatment groups, analyses including rarefaction curves, PCoA, and NMDS based on ASV levels were compared (Figure 3).

The rarefaction curves of samples from each group gradually leveled off with increasing sequencing depth, and the Coverage index approached 1.0, indicating that the sequencing depth in this study was sufficient to cover the vast majority of microbial taxa in the samples, with high data reliability (Figure 3a).

In *β*-diversity analysis, the PCoA results based on ASV levels (Figure 3b) revealed a clear separation between the NC group and the MC group in the coordinate space, suggesting that CTX treatment significantly altered the overall structure of the intestinal microbiota. Compared to the MC group, the sample points of the LBRP and HBRP groups shifted significantly and gradually moved closer to the NC group, indicating that BRP intervention effectively reversed the microbiota structural disorder induced by CTX. Notably, the HBRP group showed a higher degree of spatial overlap with the NC group, suggesting that the high dose of BRP had a more pronounced restorative effect on intestinal microbiota structure.

The NMDS analysis further validated the above findings (Figure 3c). Samples from each group displayed clear clustering trends in two-dimensional space, with the MC group significantly separated from the NC group, while the BRP intervention groups were positioned between them and exhibited a dose-dependent trend toward structural similarity. The stress value of the NMDS analysis (stress < 0.2) indicated high reliability of the ordination results.

In summary, BRP supplementation significantly modulated the overall structural abnormalities of intestinal microbiota induced by CTX, promoting its restoration toward a healthy state.

### 3.4. Effects of BRP on Gut Microbiota Diversity in CTX-Treated Mice

**Figure 4:**
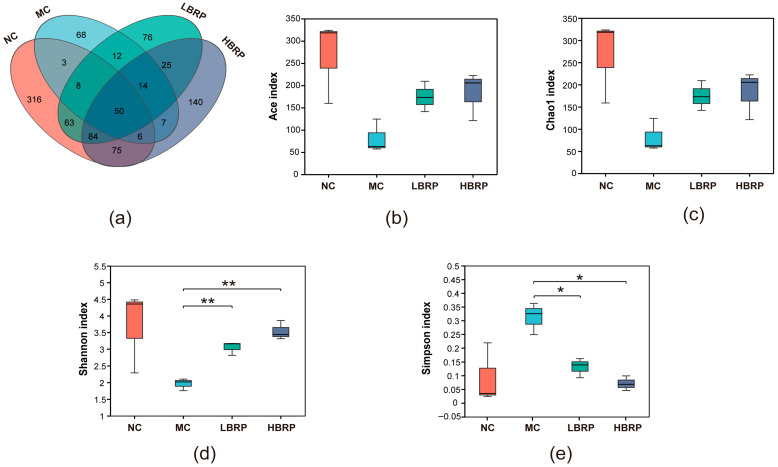
Effects of BRP on gut microbiota *α*-diversity in cyclophosphamide (CTX)-treated mice. (**a**) Venn diagram showing the number of shared and unique amplicon sequence variants (ASVs) inferred using QIIME 2’s DADA2 pipeline among the four groups. (**b**) Ace index and (**c**) Chao1 index reflecting gut microbial richness. (**d**) Shannon index and (**e**) Simpson index reflecting gut microbial diversity and evenness. Statistical analysis was performed using the Kruskal-Wallis rank-sum test followed by Games–Howell post-hoc test with false discovery rate (FDR) correction in R (version 3.3.1). * *p* < 0.05, ** *p* < 0.01.

To investigate the effects of BRP on gut microbiota composition, 16S rRNA gene sequencing was performed on fecal samples from NC, MC, LBRP, and HBRP groups (Figure S1). The Venn diagram (Figure 4a) shows the number of unique and shared amplicon sequence variants (ASVs) across the four groups. The NC group had the highest number of unique ASVs (316), followed by the HBRP group (140), LBRP group (76), and MC group (68). A total of 50 core ASVs were shared by all four groups.

Alpha diversity analysis further confirmed these findings. The Ace index (Figure 4b) and Chao1 index (Figure 4c), which reflect microbial richness, were significantly reduced in the MC group compared with the NC group, suggesting that CTX markedly decreased gut microbial abundance. BRP administration significantly restored microbial richness in a dose-dependent manner, with the HBRP group exhibiting higher Ace and Chao1 values than the LBRP group. Microbial diversity was further evaluated using the Shannon index (Figure 4d) and Simpson index (Figure 4e). CTX treatment significantly reduced the Shannon index and increased the Simpson index, indicating a loss of microbial diversity and community evenness. In contrast, both LBRP and HBRP supplementation significantly increased the Shannon index while reducing the Simpson index compared with the MC group. Notably, the HBRP group showed a more pronounced improvement in microbial diversity, approaching levels observed in the NC group.

Collectively, these results demonstrate that BRP effectively alleviates CTX-induced gut microbiota dysbiosis by restoring microbial richness and diversity, with high-dose BRP exerting stronger modulatory effects.

### 3.5. Effects of BRP on Gut Microbiota Composition at the Phylum and Genus Levels

**Figure 5:**
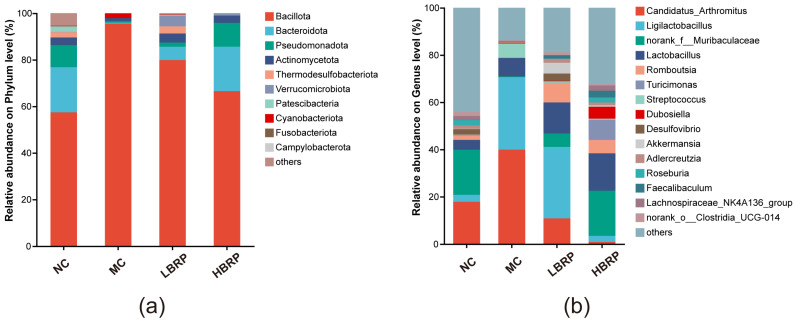
Effects of BRP on gut microbiota composition at the phylum and genus levels in CTX-treated mice. (**a**) Relative abundance of gut microbiota at the phylum level in the NC, MC, LBRP, and HBRP groups. (**b**) Relative abundance of dominant bacterial genera among different treatment groups.

To further explore the regulatory effect of *Brassica rapa* L. polysaccharides (BRP) on intestinal microbiota imbalance induced by CTX, the relative abundance of intestinal microbiota at the phylum and genus levels in each group of mice was analyzed (Figure 5).

At the phylum level (Figure 5a), the intestinal microbiota of the NC group was mainly composed of Bacillota and Bacteroidota. CTX treatment (MC group) significantly altered the microbiota structure, characterized by a significant increase in the relative abundance of Bacillota and a significant decrease in Bacteroidota, indicating that CTX induced a marked imbalance of the microbiota. Compared with the MC group, BRP intervention significantly improved the microbiota composition disorder. In the LBRP and HBRP groups, the relative abundance of Bacteroidota was significantly restored, while the abnormal proliferation of Bacillota was suppressed. Among them, the microbiota structure of the HBRP group was closer to that of the NC group.

At the genus level (Figure 5b), the relative abundance of potentially pathogenic or stress-related genera (such as *Candidatus_Arthromitus*) increased in the MC group, while the abundance of several beneficial genera significantly decreased. After BRP supplementation, the microbiota structure underwent significant remodeling. In the LBRP and HBRP groups, the relative abundance of beneficial genera such as *Ligilactobacillus*, *Lactobacillus*, *Akkermansia*, *Roseburia*, and *Faecalibaculum* significantly increased. Additionally, the HBRP group exhibited a stronger regulatory effect, with its microbiota distribution pattern more closely resembling that of the normal control group.

In summary, BRP could significantly reverse CTX-induced intestinal microbiota disorder at both the phylum and genus levels, with the high-dose intervention showing more pronounced effects.

## 4. Discussion

*Brassica rapa* L., a biennial herbaceous plant of the family Brassicaceae, has long been utilized in traditional Uyghur medicine for treating respiratory ailments such as cough and asthma, underscoring its ethnomedicinal relevance and potential pharmacological value. The Brassicaceae family, comprising a wide array of species including economically important genera such as Brassica, is known for its diverse bioactive constituents [38]. Cyclophosphamide (CTX), as a widely used chemotherapy and immunosuppressive drug, inevitably causes severe damage to the intestinal mucosal barrier and the gut microecosystem while targeting rapidly dividing cells. Nevertheless, despite the established nutritional and chemopreventive properties of Brassica vegetables, the specific protective role of BRP against chemotherapy-induced intestinal mucosal injury remains insufficiently characterized [39,40,41]. This study systematically evaluated the protective effects of *Brassica rapa* L. polysaccharides (BRP) against CTX-induced intestinal injury, providing an in-depth exploration from multiple perspectives including overall phenotype, oxidative stress, immune inflammation, and intestinal microbiota structure.

At the level of overall physiological status and histology, CTX significantly inhibited body weight gain in mice and caused severe damage to the intestinal mucosal structure, manifested as villus atrophy, disorganized arrangement, and inflammatory cell infiltration. This is consistent with previous findings on CTX-induced intestinal toxicity [42]. BRP intervention, particularly at a high dose, markedly alleviated the trend of weight loss and mitigated intestinal mucosal histopathological damage, suggesting its significant protective effect against CTX-induced intestinal injury.

CTX exposure led to marked impairment of antioxidant defense systems and increased lipid peroxidation, accompanied by hepatic dysfunction, Oxidative stress is considered one of the primary mechanisms underlying CTX-induced intestinal toxicity [43]. CTX metabolism generates excessive reactive oxygen species (ROS), which can induce lipid peroxidation, disrupt epithelial integrity, and aggravate inflammatory responses [44]. In this study, CTX treatment markedly reduced the activities of antioxidant enzymes, including SOD, CAT, and GSH-Px, while significantly increasing MDA levels, indicating severe oxidative damage. BRP supplementation significantly restored antioxidant enzyme activities and reduced MDA accumulation, particularly in the high-dose group, suggesting that BRP possesses strong free radical scavenging and antioxidative properties. Previous studies have demonstrated that natural polysaccharides can enhance endogenous antioxidant defense systems by activating antioxidant-related signaling pathways and reducing ROS generation [45,46]. The antioxidant effect of BRP may therefore contribute directly to maintaining intestinal epithelial integrity and protecting intestinal barrier function during chemotherapy.

In addition to oxidative stress, immune suppression is another hallmark of CTX-induced toxicity [47]. The intestine functions as both a digestive and immune organ, and disruption of immune homeostasis can further aggravate intestinal mucosal injury [48]. In the present study, CTX significantly reduced the levels of multiple cytokines associated with innate and adaptive immunity, including IL-1β, TNF-α, IL-2, IL-4, IL-6, IL-10, and IFN-γ, indicating broad immunosuppression. BRP intervention effectively restored these cytokines, particularly in the high-dose group, demonstrating a pronounced immunomodulatory effect. Interestingly, BRP simultaneously promoted the recovery of both pro-inflammatory and anti-inflammatory cytokines, suggesting that its protective role may involve restoration of immune balance rather than simple immune activation. Previous studies have reported that plant-derived polysaccharides can regulate macrophage activation, T-cell differentiation, and cytokine secretion, thereby enhancing host immune defense and tissue repair [49,50]. The recovery of IL-10 observed in this study may also contribute to suppression of excessive inflammatory injury and maintenance of intestinal immune homeostasis.

Accumulating evidence indicates that gut microbiota dysbiosis plays a central role in chemotherapy-induced intestinal injury [51,52]. In this study, CTX markedly reduced microbial diversity and altered the composition of dominant bacterial taxa, confirming severe disruption of intestinal microecology. BRP supplementation significantly improved *α*-diversity indices and shifted microbial community structure toward that of the normal control group, indicating restoration of microbial homeostasis. At the phylum level, CTX induced excessive enrichment of Bacillota and depletion of Bacteroidota and Pseudomonadota, whereas BRP partially reversed these alterations. Changes in the Bacillota/Bacteroidota ratio are commonly associated with intestinal inflammation, metabolic imbalance, and impaired barrier function [53,54]. Restoration of Bacteroidota abundance following BRP intervention may therefore contribute to improved intestinal metabolism and immune regulation.

At the genus level, BRP markedly reduced the abundance of *Candidatus_Arthromitus* and restored beneficial bacteria such as *Ligilactobacillus*, norank_f__Muribaculaceae, *Lactobacillus*, *Akkermansia*, and *Roseburia*. These beneficial bacteria are closely associated with intestinal barrier maintenance, anti-inflammatory activity, and short-chain fatty acid (SCFA) production [55,56]. SCFAs, particularly butyrate, serve as major energy sources for intestinal epithelial cells and play critical roles in regulating mucosal immunity and maintaining epithelial integrity [57,58]. Increased abundance of SCFA-producing bacteria following BRP supplementation suggests that BRP may improve intestinal health partly through modulation of microbial metabolites. In contrast, overgrowth of potentially harmful bacteria during CTX treatment may aggravate inflammation and oxidative damage [59]. Therefore, BRP-mediated restoration of microbial balance likely represents an important mechanism underlying its protective effects.

Notably, oxidative stress, immune responses, and gut microbiota are highly interconnected biological systems [60]. Improved antioxidant capacity may help create a favorable intestinal microenvironment for beneficial microbial colonization [61], whereas restoration of gut microbiota can further enhance host immune regulation and metabolic homeostasis through microbial metabolites and host-microbe interactions [62]. The coordinated regulation of these pathways may explain the dose-dependent protective effects observed in the present study. High-dose BRP consistently showed stronger efficacy in improving antioxidant indices, immune parameters, and microbiota composition, suggesting that sufficient polysaccharide supplementation is necessary to achieve optimal protective activity.

Overall, this study demonstrates that BRP effectively alleviates CTX-induced intestinal mucosal injury through synergistic regulation of oxidative stress, immune function, and gut microbiota homeostasis. These findings highlight the potential of BRP as a natural functional polysaccharide for preventing chemotherapy-associated intestinal toxicity. Nevertheless, the present study still has several limitations. Comprehensive structural characterization of BRP (monosaccharide composition, molecular weight, glycosidic linkages) was not performed due to limited resources. The precise molecular signaling pathways involved in BRP-mediated protection remain unclear, and causal relationships between microbiota alteration and host protection require further validation. Future studies should focus on microbiota-dependent mechanisms using germ-free or antibiotic-treated models and further investigate the role of microbial metabolites such as short-chain fatty acids in mediating the beneficial effects of BRP.

## 5. Conclusions

This study systematically evaluated the protective effects of *Brassica rapa* L. polysaccharide (BRP) in a cyclophosphamide (CTX)-induced mouse model of intestinal injury and gut microbiota dysbiosis. Overall, BRP supplementation, particularly at high dose, effectively alleviated CTX-induced physiological damage, as evidenced by improved body weight gain and restoration of intestinal histological integrity. Mechanistically, BRP significantly attenuated oxidative stress by reducing MDA levels while enhancing antioxidant enzyme activities (SOD, CAT, and GSH-Px), and concurrently modulated immune responses by suppressing pro-inflammatory cytokines (IL-1β, IL-6, TNF-α) and increasing anti-inflammatory IL-10, indicating a coordinated antioxidant-immunoregulatory protective effect. In addition, BRP reshaped gut microbiota composition and diversity, reversing CTX-induced dysbiosis by restoring microbial alpha diversity and promoting a shift in community structure, including suppression of potentially pathogenic bacteria, such as *Candidatus_Arthromitus* and *Streptococcus*, and enrichment of beneficial taxa, including *Lactobacillus* and Muribaculaceae. These microbiota changes were closely associated with immune improvement and enrichment of short-chain fatty acid-producing bacteria. Collectively, future studies should further clarify the causal role of specific microbial metabolites such as short-chain fatty acids and validate the gut microbiota-dependent mechanisms using germ-free or antibiotic-treated models, which will provide deeper insight into its translational potential in clinical adjuvant therapy.

## Figures and Tables

**Table 1:** Feeding information of mice.

Groups	First Phase (Days 0–4)	Second Phase (Days 5–16)
The control group (NC)	Sterile water (300 μL)	Sterile water (300 μL)
CTX group (MC)	Cyclophosphamide (80 mg/kg bw)	Sterile water (300 μL)
The LBRP group (LBRP)	Cyclophosphamide (80 mg/kg bw)	LBRP (80 mg/kg bw)
The HBRP group (HBRP)	Cyclophosphamide (80 mg/kg bw)	HBRP (200 mg/kg bw)

## Data Availability

The original contributions presented in this study are included in the article. Further inquiries can be directed to the corresponding author.

## Supplementary Materials

The following supporting information can be downloaded at: https://www.mdpi.com/article/10.3390/microorganisms14051146/s1, Figure S1: (a) Agarose gel electrophoresis image of the sequencing samples used for microbial diversity analysis, confirming DNA quality. (b) Genus-level phylogenetic tree of the gut microbiota across the different treatment groups (NC, MC, LBRP, HBRP).

## References

[1] Cao Q., Wang G., Peng Y. (2021). A Critical Review on Phytochemical Profile and Biological Effects of Turnip (*Brassica rapa* L.). Front. Nutr..

[2] Paul S., Geng C.-A., Yang T.-H., Yang Y.-P., Chen J.-J. (2019). Phytochemical and Health-Beneficial Progress of Turnip (*Brassica rapa*). J. Food Sci..

[3] Hua H., Zhang W., Li J., Li J., Liu C., Guo Y., Cheng Y., Pi F., Xie Y., Yao W. (2021). Neuroprotection against Cerebral Ischemia/Reperfusion by Dietary Phytochemical Extracts from Tibetan Turnip (*Brassica rapa* L.). J. Ethnopharmacol..

[4] Kong X., Yin D., Zhang L., Wang J., Hamed Y.S., Wu L., Wang Y., Sun P., Bu T., Yang K. (2026). Characterization, Stability, Sustained Release, and in Vivo Anti-Fatigue Efficacy of Zein/Chitosan Nanoparticles for the Delivery of Turnip (*Brassica rapa* L.) Glucosinolates. Food Res. Int..

[5] Dejanovic G.M., Asllanaj E., Gamba M., Raguindin P.F., Itodo O.A., Minder B., Bussler W., Metzger B., Muka T., Glisic M. (2021). Phytochemical Characterization of Turnip Greens (*Brassica rapa* ssp. Rapa): A Systematic Review. PLoS ONE.

[6] Zhang M., Wang W., Li W., Wang Z., Bi K., Li Y., Wu Y., Zhao Y., Yang R., Du Q. (2025). Ultrasonic-Assisted Extraction of Polysaccharides from *Brassica rapa* L. and Its Effects on Gut Microbiota in Humanized Mice. Foods.

[7] Wang C., Zhu H., Cheng Y., Guo Y., Zhao Y., Qian H. (2023). Aqueous Extract of *Brassica rapa* L.’s Impact on Modulating Exercise-Induced Fatigue via Gut–Muscle Axis. Nutrients.

[8] Abbaoui B., Lucas C.R., Riedl K.M., Clinton S.K., Mortazavi A. (2018). Cruciferous Vegetables, Isothiocyanates, and Bladder Cancer Prevention. Mol. Nutr. Food Res..

[9] Guo W., Liu X., Guo J., Gao R., Xiang X., An X., Bai L. (2022). Polysaccharides of *Brassica rapa* L. Attenuate Tumor Growth via Shifting Macrophages to M1-like Phenotype. Phytother. Res..

[10] Cao W., Wang C., Mayhesumu X., Pan L., Dang Y., Yili A., Abuduwaili A., Mansur S. (2022). Isolation, Structural Elucidation, Antioxidant and Hypoglycemic Activity of Polysaccharides of *Brassica rapa* L.. Molecules.

[11] Niu Y., Zhao T., Liu Z., Li D., Wen D., Li B., Huang X. (2024). *Brassica rapa* L. Crude Polysaccharide Meditated Synbiotic Fermented Whey Beverage Ameliorates Hypobaric Hypoxia Induced Intestinal Damage. Food Funct..

[12] Wei R., Liu X., Wang Y., Dong J., Wu F., Mackenzie G.G., Su Z. (2021). (−)-Epigallocatechin-3-Gallate Mitigates Cyclophosphamide-Induced Intestinal Injury by Modulating the Tight Junctions, Inflammation and Dysbiosis in Mice. Food Funct..

[13] Wu J., Chen G., Chen D., Zhang H., Lv H., Wen Z. (2025). Grifola Frondosa Polysaccharides Alleviated Cyclophosphamide—Induced Intestinal Injury Based on Microbiota, Metabolite and Immune Axis Modulation. Foods.

[14] Wang J., Li M., Gao Y., Li H., Fang L., Liu C., Liu X., Min W. (2022). Effects of Exopolysaccharides from Lactiplantibacillus Plantarum JLAU103 on Intestinal Immune Response, Oxidative Stress, and Microbial Communities in Cyclophosphamide-Induced Immunosuppressed Mice. J. Agric. Food Chem..

[15] Haque P.S., Kapur N., Barrett T.A., Theiss A.L. (2024). Mitochondrial Function and Gastrointestinal Diseases. Nat. Rev. Gastroenterol. Hepatol..

[16] Xie H., Fang J., Farag M.A., Li Z., Sun P., Shao P. (2022). *Dendrobium officinale* Leaf Polysaccharides Regulation of Immune Response and Gut Microbiota Composition in Cyclophosphamide-Treated Mice. Food Chem. X.

[17] Nwako J.G., McCauley H.A. (2024). Enteroendocrine Cells Regulate Intestinal Homeostasis and Epithelial Function. Mol. Cell. Endocrinol..

[18] Peterson L.W., Artis D. (2014). Intestinal Epithelial Cells: Regulators of Barrier Function and Immune Homeostasis. Nat. Rev. Immunol..

[19] Keely S.J., Barrett K.E. (2022). Intestinal Secretory Mechanisms and Diarrhea. Am. J. Physiol.-Gastrointest. Liver Physiol..

[20] Lin S., Mukherjee S., Li J., Hou W., Pan C., Liu J. (2021). Mucosal Immunity–Mediated Modulation of the Gut Microbiome by Oral Delivery of Probiotics into Peyer’s Patches. Sci. Adv..

[21] Zhang P., Liu J., Lee A., Tsaur I., Ohira M., Duong V., Vo N., Watari K., Su H., Kim J.Y. (2024). IL-22 Resolves MASLD via Enterocyte STAT3 Restoration of Diet-Perturbed Intestinal Homeostasis. Cell Metab..

[22] Huang J., Lee H., Zhao X., Han J., Su Y., Sun Q., Shao J., Ge J., Zhao Y., Bai X. (2021). Interleukin-17D Regulates Group 3 Innate Lymphoid Cell Function through Its Receptor CD93. Immunity.

[23] Medina-Rodríguez E.M., Martínez-Raga J., Sanz Y. (2024). Intestinal Barrier, Immunity and Microbiome: Partners in the Depression Crime. Pharmacol. Rev..

[24] Talib W.H., Abed I., Raad D., Alomari R.K., Jamal A., Jabbar R., Alhasan E.O.A., Alshaeri H.K., Alasmari M.M., Law D. (2024). Targeting Cancer Hallmarks Using Selected Food Bioactive Compounds: Potentials for Preventive and Therapeutic Strategies. Foods.

[25] Zhang J., Ma Y., Chang R., Wang R., Zhang J. (2026). The Interplay of Plant Polysaccharide Structure, Gut Microbiota Metabolism, and Host Health: Mechanisms and Perspectives. Life Sci..

[26] Chen Y., Li H., Lai F., Min T., Wu H., Zhan Q. (2024). The Influence and Mechanisms of Natural Plant Polysaccharides on Intestinal Microbiota-Mediated Metabolic Disorders. Foods.

[27] Ma Q., Zhai R., Xie X., Chen T., Zhang Z., Liu H., Nie C., Yuan X., Tu A., Tian B. (2022). Hypoglycemic Effects of Lycium Barbarum Polysaccharide in Type 2 Diabetes Mellitus Mice via Modulating Gut Microbiota. Front. Nutr..

[28] Ma Y., Wei X., Peng J., Wei F., Wen Y., Liu M., Song B., Wang Y., Zhang Y., Peng T. (2024). Ephedra Sinica Polysaccharide Regulate the Anti-Inflammatory Immunity of Intestinal Microecology and Bacterial Metabolites in Rheumatoid Arthritis. Front. Pharmacol..

[29] Song W., Zhang T., Wang Y., Xue S., Zhang Y., Zhang G. (2025). Glycyrrhiza Uralensis Polysaccharide Modulates Characteristic Bacteria and Metabolites, Improving the Immune Function of Healthy Mice. Nutrients.

[30] Ren X., Hu J., Hong Y., Guo Y., Liu Q., Yang R. (2024). Extraction, Separation and Efficacy of Yam Polysaccharide. Int. J. Biol. Macromol..

[31] Sun X., Dong J., Li J., Ye M., Zhang W., Ou J. (2017). Facile Preparation of Polysaccharide Functionalized Macroporous Adsorption Resin for Highly Selective Enrichment of Glycopeptides. J. Chromatogr. A.

[32] Villegas-Hernández L.E., Dubey V.K., Acharya G., Ahluwalia B.S. (2025). Optical Super-Resolution Histology of Formalin-Fixed Paraffin-Embedded Tissue Samples: Challenges and Opportunities. Nat. Commun..

[33] Rieger J., Pelckmann L.-M., Drewes B., Nagamoto-Combs K. (2021). Preservation and Processing of Intestinal Tissue for the Assessment of Histopathology. Animal Models of Allergic Disease.

[34] Selmi S., El-Fazaa S., Gharbi N. (2015). Oxidative Stress and Alteration of Biochemical Markers in Liver and Kidney by Malathion in Rat Pups. Toxicol. Ind. Health.

[35] Favre N., Bordmann G., Rudin W. (1997). Comparison of Cytokine Measurements Using ELISA, ELISPOT and Semi-Quantitative RT-PCR. J. Immunol. Methods.

[36] Liu C., Chu D., Kalantar-Zadeh K., George J., Young H.A., Liu G. (2021). Cytokines: From Clinical Significance to Quantification. Adv. Sci..

[37] Barberán A., Bates S.T., Casamayor E.O., Fierer N. (2012). Using Network Analysis to Explore Co-Occurrence Patterns in Soil Microbial Communities. ISME J..

[38] Zhang Y., Zhang W., Zhao Y., Peng R., Zhang Z., Xu Z., Simal-Gandara J., Yang H., Deng J. (2025). Bioactive Sulforaphane from Cruciferous Vegetables: Advances in Biosynthesis, Metabolism, Bioavailability, Delivery, Health Benefits, and Applications. Crit. Rev. Food Sci. Nutr..

[39] Wang W., Wang X., Ye H., Hu B., Zhou L., Jabbar S., Zeng X., Shen W. (2016). Optimization of Extraction, Characterization and Antioxidant Activity of Polysaccharides from *Brassica rapa* L.. Int. J. Biol. Macromol..

[40] Pedras M.S.C., Yaya E.E. (2010). Phytoalexins from Brassicaceae: News from the Front. Phytochemistry.

[41] Li Z., Lee H.W., Liang X., Liang D., Wang Q., Huang D., Ong C.N. (2018). Profiling of Phenolic Compounds and Antioxidant Activity of 12 Cruciferous Vegetables. Molecules.

[42] Xuan J., Bao S., Guo L., Jiang B., Wang J., Ren D. (2026). *Cordyceps militaris* Exopolysaccharides and *Lactiplantibacillus plantarum* H8 Synergistically Improve Immune Function Damage in Cyclophosphamide-Induced Immunosuppressed Mice. Probiotics Antimicro. Prot..

[43] Feng Y., Zhang W., Xu X., Wang W., Xu Y., Wang M., Zhang J., Xu H., Fu F. (2024). Protective Effect of Luffa Cylindrica Fermentation Liquid on Cyclophosphamide-Induced Premature Ovarian Failure in Female Mice by Attenuating Oxidative Stress, Inflammation and Apoptosis. J. Ovarian Res..

[44] Zhao Q., Wang J., Liang H., Guo W., Chu Y., Liu L., Kang W. (2024). Prevention of Cyclophosphamide-Induced Immune Suppression by Polysaccharides from Apocynum Venetum Flowers via Enhancing Immune Response, Reducing Oxidative Stress, and Regulating Gut Microbiota in Mice. Front. Pharmacol..

[45] Huang L., Shen M., Wu T., Yu Y., Yu Q., Chen Y., Xie J. (2020). *Mesona chinensis* Benth Polysaccharides Protect against Oxidative Stress and Immunosuppression in Cyclophosphamide-Treated Mice via MAPKs Signal Transduction Pathways. Int. J. Biol. Macromol..

[46] Xing Y., Zheng Y., Chen L., Xu Y., Jin X., Hong L., Yan S., Shi B. (2024). Artemisia Ordosica Polysaccharides Enhance Antioxidant Capacity of Peripheral Blood Lymphocytes in Poultry Through Nrf2/Keap1 and TLR4/NF-κB Signal Pathway. Antioxidants.

[47] Li M., Wang J., Huo B., Wan Q., Xing L., Wang Y., Pei H., Wang L., Xia Y., Cui H. (2025). Umbelliferone Enhances Immune Function in Cyclophosphamide-Induced Immunosuppressed Mice via Histidine and Purine Metabolism Regulation. Curr. Drug Metab..

[48] Tian W., Peng S., Xu F., Kong X., Li Y., Zhang W., Liu Z., Zhang H., Wang J., Wang L. (2026). Disruption of Intestinal Barrier and Immune Homeostasis Links Gut Microbiota Dysbiosis to Aggravated Experimental Autoimmune Myasthenia Gravis. Front. Cell. Infect. Microbiol..

[49] Yang Y., Chen J., Lei L., Li F., Tang Y., Yuan Y., Zhang Y., Wu S., Yin R., Ming J. (2019). Acetylation of Polysaccharide from *Morchella angusticeps* Peck Enhances Its Immune Activation and Anti-Inflammatory Activities in Macrophage RAW264.7 cells. Food Chem. Toxicol..

[50] Lin J., Wang L., Li W., Li Y., Tang F., Xu J., Li W., Gong H., Jiang X., Feng Y. (2024). Dried Tangerine Peel Polysaccharide Accelerates Wound Healing by Recruiting Anti-Inflammatory Macrophages. Int. Immunopharmacol..

[51] Wang F., Li Y., Feng X., Shu Y., Zhang C., Li L., Guo M. (2026). Gut Microbiota and Metabolomic: The Potential Mechanism Underlying the Female-Specific Protective Effects of *Craterellus cornucopioides* Polysaccharide against Chemotherapy-Induced Intestinal Injury. Int. J. Biol. Macromol..

[52] Peng W., Fan X., Shi H., Jiang Y., Fan L., Xing Y., Peng Y., He Y., Zou W., Jiang M. (2025). Gut Microbiota and Chemotherapy-Induced Gastrointestinal Toxicity: Mechanisms and Intervention Strategies. Dig. Dis..

[53] Yañez C.M., Hernández A.M., Sandoval A.M., Domínguez M.A.M., Muñiz S.A.Z., Gómez J.O.G. (2021). Prevalence of Blastocystis and Its Association with Firmicutes/Bacteroidetes Ratio in Clinically Healthy and Metabolically Ill Subjects. BMC Microbiol..

[54] Jacob T., Sindhu S., Hasan A., Malik M.Z., Arefanian H., Al-Rashed F., Nizam R., Kochumon S., Thomas R., Bahman F. (2024). Soybean Oil-Based HFD Induces Gut Dysbiosis That Leads to Steatosis, Hepatic Inflammation and Insulin Resistance in Mice. Front. Microbiol..

[55] Liu A., Wang B., Wang M., Tang R., Xu W., Xiao W. (2025). L-Theanine Alleviates Ulcerative Colitis by Repairing the Intestinal Barrier through Regulating the Gut Microbiota and Associated Short-Chain Fatty Acids. Food Chem. Toxicol..

[56] Ottria R., Mirmajidi S., Ciuffreda P. (2026). Gut Microbiota-Derived Short-Chain Fatty Acids in Inflammatory Bowel Disease: Mechanistic Insights into Gut Inflammation, Barrier Function, and Therapeutic Potential. Int. J. Mol. Sci..

[57] Li W., Chen D., Zhu Y., Ye Q., Hua Y., Jiang P., Xiang Y., Xu Y., Pan Y., Yang H. (2024). Alleviating Pyroptosis of Intestinal Epithelial Cells to Restore Mucosal Integrity in Ulcerative Colitis by Targeting Delivery of 4-Octyl-Itaconate. ACS Nano.

[58] Liu M., Lu Y., Xue G., Han L., Jia H., Wang Z., Zhang J., Liu P., Yang C., Zhou Y. (2024). Role of Short-Chain Fatty Acids in Host Physiology. Anim. Models Exp. Med..

[59] Zhang Y., Wang H., Ge Q., Shi J., Zhang H., Gao J., Han J. (2025). Polysaccharides of *Melientha longistaminea* Regulates Immune Function and Gut Microbiota in Cyclophosphamide (CTX)-Induced Immunosuppressed Mice. Int. Immunopharmacol..

[60] Gu X.-M., Ye D.-C., Cheng J.-W., Lin Z.-X., Lv H.-X., Cai Z.-X., Yang Y., Wang C.-K., Gao Y.-Y., Jin L. (2025). Astaxanthin Enhances Broiler Health via Integrated Regulation of Antioxidant Capacity, Immune Function, and Gut Microbiota. Food Chem..

[61] Huang Q.-X., Liang J.-L., Yang C.-H., Li K., Niu M.-T., Fan J.-X., Zhang X.-Z. (2023). Stimulation-Responsive Mucoadhesive Probiotics for Inflammatory Bowel Disease Treatment by Scavenging Reactive Oxygen Species and Regulating Gut Microbiota. Biomaterials.

[62] Wu J., Wang K., Wang X., Pang Y., Jiang C. (2021). The Role of the Gut Microbiome and Its Metabolites in Metabolic Diseases. Protein Cell.

