# Supplementary material for: Brassica rapa L. Polysaccharides Alleviate Cyclophosphamide-Induced Intestinal Mucosal Injury in Mice by Modulating Oxidative Stress, Immune Responses, and Gut Microbiota
Source: Microorganisms. 2026 May 19;14(5):1146. doi: 10.3390/microorganisms14051146 (PMC13209187; doi:10.3390/microorganisms14051146)
Supplement: Supplementary file 1 [file microorganisms-4279981-supplementary.pdf › microorganisms-4279981-supplementary.pdf]

(a)

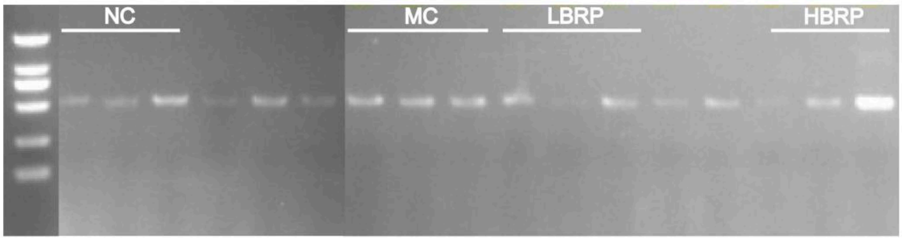

(b)

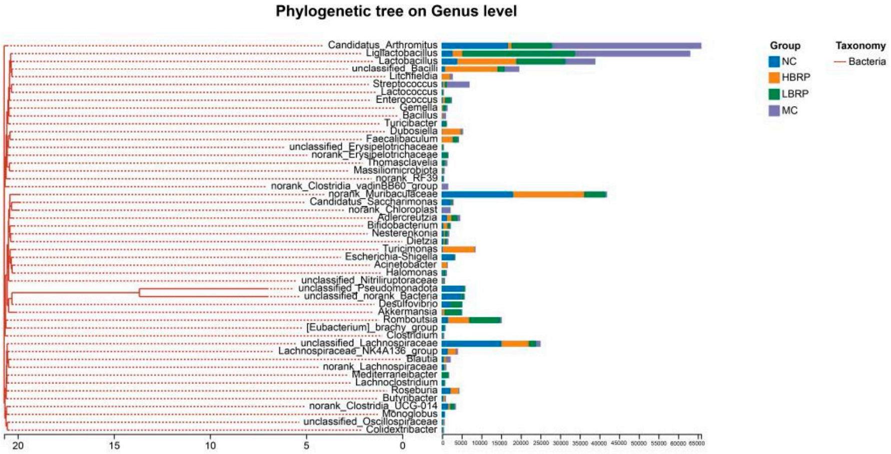

Figure S1. (a) Agarose gel electrophoresis image of the sequencing samples used for microbial diversity analysis, confirming DNA quality. (b) Genus-level phylogenetic tree of the gut microbiota across the different treatment groups (NC, MC, LBRP, HBRP).
